# Sport-related neurocognitive performance and repetitive head-impact exposure in elite Muay Thai athletes: a matched athletic-control study

**DOI:** 10.3389/fspor.2026.1908733

**Published:** 2026-10-14

**Authors:** Raja Syed Tengku Sulaiman

**Affiliations:** Major in Physical Education, School of Education, Walailak University, Nakhon Si Thammarat, Thailand

**Keywords:** combat sports, executive function, Muay Thai, repetitive head impacts, sport-related neurocognitive performance

## Abstract

**Background:**

Repetitive head impacts (RHIs) are increasingly studied in combat sports, but evidence regarding neurocognitive performance in elite Muay Thai athletes remains limited. This study compared neurocognitive performance between elite Muay Thai athletes and sprint-athlete controls and explored associations between cumulative RHI exposure and neurocognitive outcomes within the Muay Thai cohort.

**Methods:**

This cross-sectional athletic-control study included 44 elite male Muay Thai athletes and 42 male sprint-athlete controls aged 15–25 years. Controls were selected for group-level comparability in age, education, training years, and competitive level. Neurocognitive performance was assessed using the Rey Auditory Verbal Learning Test, Go/No-Go task, Stroop Colour–Word Test, Trail Making Test Parts A and B, Digit Span, and Symbol Digit Modalities Test. Task-specific outcomes and standardised domain-level indicators were examined using independent-group comparisons and covariate-adjusted ANCOVA models. Exploratory Spearman correlations examined associations between a cumulative RHI exposure score derived from self-reported proxy indicators and neurocognitive outcomes within the Muay Thai cohort.

**Results:**

No significant between-group differences were observed in task-specific or domain-level neurocognitive outcomes (all *p* > 0.05), and adjusted ANCOVA models showed no significant group effects across the four domain-level indicators. Within the Muay Thai cohort, cumulative RHI exposure was associated with the executive-function composite (*ρ* = −0.32, *p* = 0.036), TMT-A completion time (*ρ* = −0.44, *p* = 0.003), TMT-B completion time (*ρ* = −0.41, *p* = 0.006), and Stroop interference time (*ρ* = 0.56, *p* < 0.001). No other associations were significant.

**Conclusions:**

Elite Muay Thai athletes did not demonstrate significant group-level neurocognitive differences compared with sprint-athlete controls. Exploratory associations between cumulative RHI exposure and selected neurocognitive outcomes were observed within the Muay Thai cohort but were not consistent across the broader neurocognitive battery. These findings should not be interpreted as evidence of generalised neurocognitive impairment. Given the cross-sectional design, retrospective proxy-based exposure assessment, and exploratory correlations unadjusted for multiple comparisons, the findings are hypothesis-generating rather than causal or confirmatory. Prospective longitudinal studies incorporating objective head-impact monitoring and repeated neurocognitive assessment are needed.

## 1 Introduction

Muay Thai is a striking combat sport in which punches, elbows, knees, and kicks to the head may occur during both training and competition. Sport-related concussion is an acute brain injury resulting from biomechanical forces transmitted to the head, neck, or body and is associated with transient neurological dysfunction. In contrast, repetitive head impacts (RHIs) refer to repeated head-impact exposures that may occur with or without immediately apparent clinical symptoms; impacts that do not result in clinically recognised concussion are commonly termed subconcussive exposures (1). This distinction is particularly relevant to combat sports, where striking-related RHIs may accumulate across training sessions and competitive bouts.

Evidence from contact and collision sports indicates that greater RHI exposure can be associated with specific brain-structural and cognitive findings, although these findings are not uniform across populations or outcome measures. In amateur soccer players, greater heading exposure was associated with lower fractional anisotropy in temporo-occipital white-matter regions, and lower fractional anisotropy was associated with poorer memory performance (2). More recently, in adult amateur soccer players, greater RHI exposure was associated with attenuation of the fractional-anisotropy slope at the orbitofrontal gray–white matter interface and poorer verbal-learning performance. However, other measures in the same assessment battery, including delayed recall, working memory, processing speed, and executive function, were not associated with soccer RHI exposure (3). Among former American football players, white-matter perivascular-space volume was larger than in unexposed controls and showed associations with estimated cumulative RHI exposure and cognitive performance (4). These findings illustrate outcome specificity: in these studies, RHI exposure was associated with selected neuroimaging and cognitive measures, whereas other measures within the same populations showed no corresponding association. Therefore, findings from one sport, exposure metric, or assessment modality should not be assumed to represent a uniform neurocognitive consequence of RHI exposure.

Combat-sport studies likewise provide mixed but increasingly specific evidence. In boxers, comparisons of less experienced and more experienced athletes using resting electroencephalography identified lower normalised power spectral density and power-law exponent values in the more experienced group, together with frequency-dependent differences in multiscale entropy (5). Because these athletes were asymptomatic, the reported electrophysiological differences were interpreted as potential correlates of cumulative subconcussive exposure rather than evidence of cognitive impairment. In mixed martial arts athletes, reduced N400 event-related-potential amplitude relative to matched controls has also been reported, suggesting altered neural processing in the absence of clinically diagnosed concussion (6). By contrast, longitudinal neuropsychological evidence indicates that competitive mixed martial arts fighters may demonstrate poorer cognitive trajectories than recreational fighters: over two years, competitive fighters showed greater declines in mental processing speed, inhibitory control, and cognitive flexibility, together with poorer performance on additional controlled-processing and memory measures (7). Collectively, these findings suggest that repetitive head-impact exposure in combat sports may be associated with electrophysiological alterations and, in some contexts, poorer neurocognitive performance. However, electrophysiological differences should not be interpreted as evidence of impairment on behavioural cognitive tests without direct supporting evidence.

Martial-arts and other forms of athletic training have been examined in relation to executive control, inhibitory processing, attention, and motor coordination (8–10). More broadly, regular physical activity has been associated with better executive-function performance, with meta-analytic evidence indicating potential benefits for executive function, memory, and processing speed, although effect magnitudes vary according to exercise characteristics, population, and cognitive outcome (11–13). Consequently, poorer, equivalent, or better cognitive-test performance in combat-sport athletes relative to comparison groups cannot readily be attributed to head-impact exposure alone because sustained athletic training may independently influence the same cognitive domains under investigation.

This problem is evident in research involving athletic populations with differing head-impact exposure. Studies have not consistently demonstrated poorer behavioural performance with greater exposure. For example, collegiate collision-sport career duration was not associated with single- or dual-task gait performance, despite longer participation representing greater opportunity for RHI exposure (14). Conversely, longitudinal mixed martial arts data indicated progressive declines in processing speed, inhibitory control, and cognitive flexibility among competitive fighters relative to recreational fighters (7). Soccer research likewise demonstrates outcome specificity: greater heading exposure has been associated with poorer memory or verbal-learning performance, whereas other cognitive tasks within the same assessment batteries have shown no corresponding association (2, 3). Taken together, these contrasting findings suggest that potential RHI-related cognitive differences may depend on exposure characteristics, comparator selection, and the outcome assessed rather than producing a uniform global deficit. Differences in exposure quantification, test selection, task familiarity, fatigue, and correlations among cognitive outcomes further complicate cross-study interpretation (14–16). A closely matched athletic-control design may therefore provide a more informative framework for examining whether athletes routinely exposed to striking-related RHIs perform differently from comparably trained athletes without routine head-impact exposure.

The present cross-sectional study used a matched athletic-control design to compare neurocognitive performance between elite Muay Thai athletes and sprint-athlete controls. Sprint athletes were selected as a highly trained comparison group without routine exposure to striking-related head impacts, thereby reducing potential confounding by characteristics associated with sustained athletic participation (14, 15, 17). The multidimensional assessment battery evaluated verbal learning and memory, inhibitory control and attention, executive function and cognitive flexibility, processing speed, and working memory using established task-specific measures (18–24).

The primary aim was to determine whether task-specific and domain-level neurocognitive performance differed between elite Muay Thai athletes and sprint-athlete controls selected for group-level comparability. Based on evidence of poorer performance on selected processing-speed, inhibitory-control, cognitive-flexibility, and memory-related measures among athletes with greater or more sustained RHI exposure, we hypothesised that elite Muay Thai athletes would demonstrate poorer neurocognitive performance than sprint-athlete controls, particularly on executive-function and processing-speed measures. The secondary, exploratory aim was to examine associations between cumulative RHI exposure and task-specific and domain-level neurocognitive outcomes within the Muay Thai cohort.

## 2 Materials and methods

### 2.1 Study design and ethical approval

The present study used a cross-sectional, matched athletic-control design. The study was conducted in accordance with the Declaration of Helsinki (25) and was approved by the Human Research Ethics Committee of Walailak University, Thailand (WUEC-25-130-01). All adult participants provided written informed consent before participation. For participants aged <18 years, written informed consent was obtained from a parent or legal guardian, and written assent was obtained from the participant.

### 2.2 Participants and recruitment

Male athletes aged 15–25 years were recruited from Southern Thailand. Muay Thai athletes were recruited from professional Muay Thai camps and high-performance combat-sport centres, whereas sprint-athlete controls were recruited from athletics programmes and university sprint-training environments within the same geographical region. Participant recruitment, exclusions, and inclusion in the final analytical sample are shown in Figure 1. Sprint athletes were selected as the athletic-control group because they undertook structured training and competition but were not routinely exposed to striking-related head impacts. This comparison was intended to reduce potential confounding associated with general athletic participation in between-group neurocognitive comparisons (14, 15, 17).

**Figure 1:**
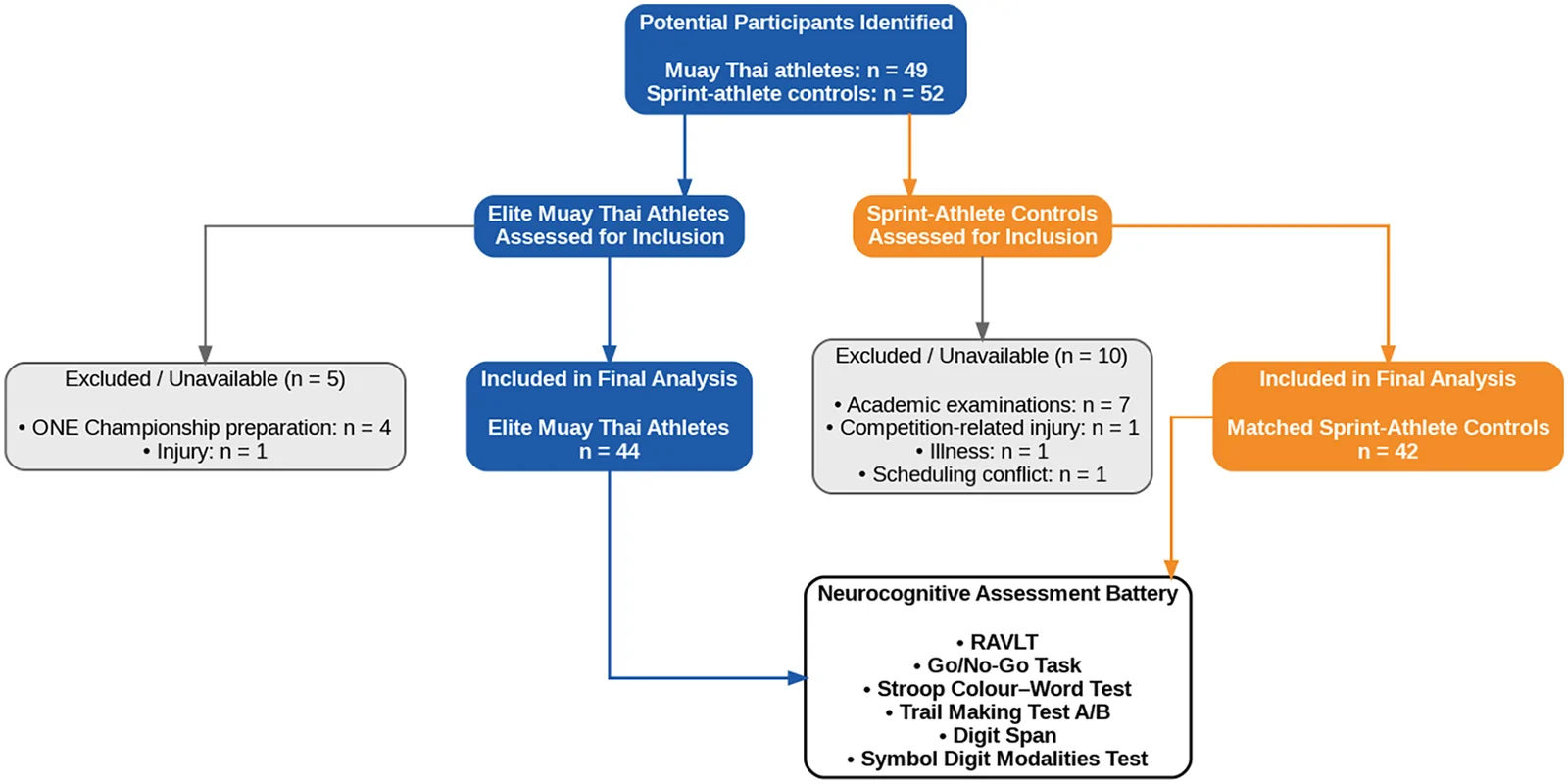
Flow diagram illustrating participant recruitment, exclusions, group-level matching procedures, and final analytical inclusion of elite Muay Thai athletes and sprint-athlete controls. A total of 44 elite Muay Thai athletes and 42 sprint-athlete controls selected to achieve group-level comparability in age, educational attainment, years of structured training, and competitive level completed the multidimensional neurocognitive assessment battery and were included in the final analyses.

### 2.3 Eligibility criteria and athletic-control matching

Muay Thai athletes were eligible if they were male, aged 15–25 years, had competed at the Thailand National Games or Thailand National Youth Games level, had completed ≥3 years of structured Muay Thai training, and trained regularly ≥4 sessions per week. Sprint-athlete controls were eligible if they were male, aged 15–25 years, competed at national or regional level, and had completed ≥3 years of structured sprint training.

Participants in either group were excluded if they reported a current or previous neurological disorder; a history of moderate-to-severe traumatic brain injury; a current musculoskeletal injury that could interfere with testing; a psychiatric or neurodevelopmental disorder requiring ongoing treatment; current use of medication considered likely to influence cognitive performance; or uncorrected visual impairment that could interfere with neurocognitive testing. Medical and injury histories, medication use, and visual status were obtained by participant self-report using a structured health-screening questionnaire before testing.

Sprint-athlete controls were purposively selected to achieve group-level comparability with the Muay Thai cohort with respect to age, educational attainment, years of structured training, and competitive level. Matching was performed at the group level rather than through individual one-to-one matching, and these characteristics guided control selection. Between-group comparisons of matching variables and other participant characteristics are reported in Table 1.

**Table 1:** Participant characteristics of elite Muay Thai athletes and sprint-athlete controls (mean ± SD).

Variable	Muay Thai (*n* = 44)	Sprinter (*n* = 42)	*p*-value	Hedges g	Test
Age	20.23 ± 2.84	19.93 ± 2.90	0.620	0.10	Mann–Whitney U
Height	172.36 ± 4.55	170.83 ± 3.27	0.076	0.38	Welch t-test
Body mass	58.05 ± 5.64	56.23 ± 3.74	0.081	0.38	Welch t-test
Training years	4.84 ± 1.57	4.71 ± 1.70	0.531	0.08	Mann–Whitney U
Competition exposure	25.00 ± 4.31	24.79 ± 4.29	0.818	0.05	Welch t-test
Education years	13.86 ± 2.88	13.40 ± 2.72	0.492	0.16	Mann–Whitney U

Data are presented as mean ± standard deviation (SD). Positive Hedges’ *g* values indicate higher mean values in elite Muay Thai athletes relative to sprint-athlete controls. Welch independent-samples *t*-tests were applied to approximately normally distributed variables, whereas Mann–Whitney *U* tests were used when normality assumptions were not satisfied.

### 2.4 Procedures and data collection

All assessments were conducted in a quiet indoor environment under standardised testing conditions. Participants were instructed to avoid strenuous exercise, alcohol consumption, and caffeine for at least 24 h before testing (11, 26). Testing was conducted during daytime hours. Before formal assessment, participants received standardised instructions and were familiarised with the testing procedures. Scheduled rest periods were provided during the assessment session to minimise cognitive fatigue and potential order-related performance effects (16).

Before neurocognitive testing, participant characteristics and relevant health, injury, training, and competition histories were collected using structured data-recording procedures. Within the Muay Thai cohort, additional information on previous head-injury events was obtained through an interviewer-administered structured interview. Participants reported the number of previous head-injury events, their age at the time of each reported event, and associated symptoms. Training and competition information relevant to cumulative RHI exposure, including years of structured Muay Thai training and number of competitive fights, was also recorded. These retrospective data were subsequently used to characterise cumulative repetitive head-impact (RHI) exposure, as described in Section 2.6.

Following data collection and familiarisation, participants completed the multidimensional neurocognitive assessment battery described in Section 2.5.

### 2.5 Neurocognitive assessment battery

Sport-related neurocognitive performance was assessed using a multidimensional battery comprising the Rey Auditory Verbal Learning Test (RAVLT), a computerised Go/No-Go task, the Stroop Colour–Word Test, Trail Making Test Parts A and B (TMT-A and TMT-B), Digit Span, and the Symbol Digit Modalities Test (SDMT) (18–24). Collectively, the battery evaluated verbal learning and memory, inhibitory control, sustained attention, executive function, cognitive flexibility, processing speed, visuomotor scanning, and working memory.

The RAVLT assessed verbal learning and memory. Immediate recall, delayed recall, recognition, and interference recall were retained as task-specific outcomes, with higher scores indicating better performance (20, 27, 28).

The computerised Go/No-Go task was administered using Inquisit Lab version 7.1.3 (Millisecond Software, LLC, Seattle, WA, USA) to assess inhibitory control and sustained attention. The paradigm followed established Go/No-Go approaches to response inhibition, in which frequent Go trials establish a prepotent response tendency and less frequent No-Go trials require response withholding (29–31). Visual stimuli comprised green and red circles, each 50 mm in diameter, presented individually against a black background. Participants were instructed to respond as quickly and accurately as possible when a green circle (Go stimulus) appeared and to withhold their response when a red circle (No-Go stimulus) appeared. The task comprised 360 trials, including 270 Go trials (75%) and 90 No-Go trials (25%), delivered in smaller blocks to minimise fatigue. Correct responses were followed by an on-screen “CORRECT” message, whereas incorrect responses received no feedback. Mean reaction time for correct Go trials (ms) and overall response accuracy (%) were retained as task-specific outcomes.

The Stroop Colour–Word Test assessed inhibitory control. Stroop interference time was retained as the task-specific outcome, with shorter completion times indicating better performance. TMT-A completion time was used as an indicator of processing speed and visuomotor scanning, whereas TMT-B completion time assessed executive function and cognitive flexibility. Shorter completion times indicated better performance on both TMT measures (21, 22).

The SDMT assessed processing speed, attention, and visuomotor integration. The total number of correct responses was retained as the task-specific outcome, with higher scores indicating better performance. Digit Span assessed working memory, and the total Digit Span score was retained as the task-specific outcome, with higher scores indicating better performance (23, 24).

Task-specific outcomes were retained for the primary descriptive and between-group analyses. Domain-level composite indicators were additionally derived to summarise performance across conceptually related neurocognitive measures and to complement, rather than replace, the task-specific analyses. The construction and statistical treatment of these domain-level indicators are described in Section 2.7.

### 2.6 Repetitive head-impact exposure assessment

Within the Muay Thai cohort, repetitive head-impact (RHI) exposure was estimated using information obtained through an interviewer-administered structured interview. Participants reported their Muay Thai training and competition histories and previous head-injury events sustained during training or competition. Information collected included the total number of reported head-injury events, age at occurrence, and associated symptoms, including dizziness, confusion, severe headache, nausea, blurred or double vision, or brief loss of consciousness. These events were based on retrospective participant reports and were not restricted to events that had received a formal medical diagnosis. Age at occurrence and associated symptoms were collected to characterise reported injury history but were not included in the cumulative exposure score.

Consistent with proxy-based approaches used when direct biomechanical monitoring of head impacts is unavailable (15), cumulative RHI exposure was characterised using three retrospective indicators: years of structured Muay Thai training, number of competitive fights, and self-reported number of head-injury events. Because these indicators were measured on different numerical scales, each was standardised as a *z* score within the Muay Thai cohort before combination. The cumulative RHI exposure score was calculated as:

$$\begin{array}{r} \mathrm{Cumulative}\;\mathrm{RHI}\;\mathrm{exposure}\;\mathrm{score}\;=z(\mathrm{training}\;\mathrm{years})\;\\ +\;z(\mathrm{number}\;\mathrm{of}\;\;\mathrm{competitive}\;\mathrm{fights})\;\\ +\;z(\mathrm{self}-\mathrm{reported}\;\mathrm{number}\;\mathrm{of}\;\;\mathrm{head}-\mathrm{injury}\;\mathrm{events}). \end{array}$$

Accordingly, the exposure score was based on the sum of standardised rather than raw values, placing the three indicators on a common standardised scale before combination. Higher scores represented greater estimated cumulative RHI exposure. The score was used only in exploratory within-cohort analyses.

Because the exposure indicators were retrospectively self-reported rather than derived from direct biomechanical monitoring or prospective medical surveillance, the cumulative RHI exposure score should be interpreted as a proxy measure that may be affected by recall bias and exposure misclassification. Descriptive exposure characteristics are presented in Table 5.

### 2.7 Statistical analysis

Statistical analyses were performed using SPSS version 29.0 (IBM Corp., Armonk, NY, USA). Statistical significance was set at *p* < 0.05 (two-tailed). Data are presented as mean ± standard deviation unless otherwise stated. Normality of task-specific outcomes was assessed using Shapiro–Wilk tests together with visual inspection of distributions and Q–Q plots (16). For task-specific between-group comparisons, Welch independent-samples *t*-tests were used for approximately normally distributed outcomes, whereas Mann–Whitney *U* tests were applied when distributional assumptions were not adequately met. Effect sizes for between-group comparisons were calculated using Hedges’ *g* and interpreted as trivial (<0.20), small (0.20–0.49), moderate (0.50–0.79), or large (≥0.80) (17).

In addition to task-specific outcomes, domain-level indicators were constructed to summarise performance across conceptually related neurocognitive measures representing processing speed, executive function, memory, and working memory. These indicators complemented, rather than replaced, the task-specific analyses. For construction of the domain-level indicators, measures for which lower raw values represented better performance were reverse-scored before standardisation so that higher standardised values consistently reflected better neurocognitive performance. Neurocognitive measures were standardised as *z* scores across the full analytical sample (*N* = 86) to account for differences in measurement scales. For domains represented by multiple measures, the relevant standardised scores were averaged to derive the corresponding domain-level indicator. Working memory was represented by the standardised Digit Span score. Task-specific analyses retained the original scoring direction of the individual measures; thus, TMT-A, TMT-B, and Stroop interference were analysed as raw completion times, and Go/No-Go reaction time was analysed in its original time-based direction.

Processing speed comprised reversed TMT-A completion time, SDMT performance, and reversed mean Go/No-Go reaction time. Executive function comprised reversed TMT-B completion time, reversed Stroop interference time, and Go/No-Go overall response accuracy. Memory comprised RAVLT immediate recall, delayed recall, recognition, and interference recall. This standardisation procedure was applied to neurocognitive outcomes and was distinct from the within-Muay Thai standardisation used to construct the cumulative RHI exposure score described in Section 2.6.

Because athletic-control matching was performed at the group level rather than through individual one-to-one pairing, between-group analyses were conducted as independent-group comparisons. Separate analysis of covariance (ANCOVA) models were fitted for the processing-speed, executive-function, memory, and working-memory domain-level indicators. In each model, group was entered as the fixed factor, and age, years of education, and years of sport-specific training were entered as covariates. These covariates were included to account for potential residual demographic and training-related differences after group-level matching. The ANCOVA models were interpreted as complementary adjusted analyses rather than paired analyses.

Before interpretation of the ANCOVA models, linearity between the continuous covariates and each outcome, homogeneity of regression slopes, normality of residuals, homoscedasticity, potentially influential observations, and multicollinearity were evaluated. Homogeneity of regression slopes was assessed using Group×Age, Group×Education, and Group×Training Years interaction terms. Collinearity among covariates was evaluated using pairwise correlations, tolerance values, and variance inflation factors (VIFs). Residual normality was assessed using Q–Q plots and Shapiro–Wilk tests of model residuals. Homoscedasticity was evaluated using residual-versus-fitted plots and Levene's test. Standardised residuals, leverage values, and Cook's distance were inspected to identify potentially influential observations. Diagnostic findings were considered when interpreting the adjusted models, and observations were not excluded solely on the basis of a single diagnostic criterion. Where evidence of heteroscedasticity was identified, a heteroscedasticity-consistent (HC3) covariance estimator was used as a sensitivity analysis to assess the robustness of the corresponding group effect.

Exploratory associations between cumulative RHI exposure and neurocognitive outcomes within the Muay Thai cohort were assessed using Spearman rank-order correlations. For task-specific correlations, outcomes were analysed in their original scoring directions rather than the direction-aligned scores used to construct the domain-level indicators. Spearman correlations were selected because the cumulative RHI exposure score incorporated self-reported head-injury counts with a substantial number of zero values and tied observations. This non-parametric rank-based approach was used to assess monotonic associations without requiring normally distributed variables or a linear relationship. No adjustment for multiple comparisons was applied to these exploratory analyses; therefore, reported *p*-values are unadjusted and were interpreted as hypothesis-generating rather than confirmatory.

## 3 Results

### 3.1 Participant characteristics and matching variables

Participant recruitment, exclusions, group-level matching procedures, and the final analytical sample are shown in Figure 1. The final analytical sample comprised 44 elite Muay Thai athletes and 42 sprint-athlete controls selected to achieve group-level comparability with respect to age, educational attainment, years of structured training, and competitive level.

No statistically significant between-group differences were observed in age, years of structured training, competition exposure, educational attainment, height, or body mass (all *p* > 0.05), and between-group effect sizes were generally small. Participant characteristics and corresponding between-group comparisons are presented in Table 1. Normality assessments for neurocognitive variables are provided in Supplementary Table S1.

Categorical participant characteristics, including competitive level, educational status, and medal distribution, are presented in Table 2.

**Table 2:** Categorical participant characteristics of elite Muay Thai athletes and sprint-athlete controls.

Variable	Category	Muay Thai *n* (%)	Sprinter *n* (%)
Competition level	Thailand National Games	28	25
Competition level	Thailand National Youth Games	16	17
Education level	Bachelor	6	6
Education level	High School	26	28
Education level	Master	12	8
Medal status	Bronze	13	15
Medal status	Gold	16	11
Medal status	Silver	15	16

Values represent observed category frequencies in the final analytical sample. Medal status is presented descriptively and was not included as a primary matching variable.

### 3.2 Task-specific neurocognitive outcomes

Task-specific neurocognitive outcomes are presented in Table 3. No statistically significant between-group differences were observed for TMT-A or TMT-B completion time, Stroop interference time, Go/No-Go reaction time or response accuracy, SDMT performance, Digit Span performance, or RAVLT immediate recall, delayed recall, recognition, or interference recall (all *p* > 0.05).

**Table 3:** Raw neurocognitive outcomes for elite Muay Thai athletes and sprint-athlete controls (mean ± SD).

Variable	Muay Thai (*n* = 44)	Sprinter (*n* = 42)	*p*-value	Hedges g	Test
TMT-A	17.64 ± 0.90	17.74 ± 1.08	0.634	−0.10	Welch t-test
TMT-B	52.83 ± 1.73	52.74 ± 1.78	0.822	0.05	Welch t-test
Digit Span	22.64 ± 2.17	22.02 ± 2.99	0.339	0.23	Mann–Whitney U
Stroop interference time	65.02 ± 2.02	64.50 ± 2.62	0.305	0.22	Welch t-test
SDMT	63.61 ± 3.69	64.21 ± 3.20	0.490	−0.17	Mann–Whitney U
RAVLT Immediate Recall	13.07 ± 1.07	13.45 ± 0.92	0.177	−0.38	Mann–Whitney U
RAVLT Delayed Recall	12.07 ± 1.21	12.31 ± 1.22	0.538	−0.20	Mann–Whitney U
RAVLT Recognition	12.39 ± 1.20	12.74 ± 1.25	0.259	−0.28	Mann–Whitney U
RAVLT Interference Recall	12.93 ± 1.11	13.31 ± 1.16	0.169	−0.33	Mann–Whitney U
Reaction time	224.43 ± 10.64	224.17 ± 10.81	0.909	0.02	Welch t-test
Response accuracy	95.18 ± 2.36	95.21 ± 2.52	0.951	−0.01	Welch t-test

Data are presented as mean ± SD. Comparisons are unadjusted. Higher reaction-time and Trail Making Test (TMT) scores indicate slower task performance. Hedges’ *g* values are reported as elite Muay Thai athletes minus sprint-athlete controls. TMT, Trail Making Test; SDMT, Symbol Digit Modalities Test; RAVLT, Rey Auditory Verbal Learning Test.

### 3.3 Composite neurocognitive domain scores

Composite neurocognitive-domain indicators are presented in Table 4 and illustrated in Figure 2. No statistically significant between-group differences were observed for processing speed, executive function, memory, or working memory (all *p* > 0.05).

**Table 4:** Composite neurocognitive-domain scores in elite Muay Thai athletes and sprint-athlete controls (mean ± SD).

Variable	Muay Thai (*n* = 44)	Sprinter (*n* = 42)	*p*-value	Hedges *g*	Test
Processing speed composite	−0.03 ± 0.56	0.03 ± 0.56	0.653	−0.10	Welch t-test
Executive function composite	−0.01 ± 0.52	0.01 ± 0.56	0.891	−0.03	Welch t-test
Memory composite	−0.13 ± 0.74	0.14 ± 0.76	0.267	−0.36	Mann–Whitney U
Working memory composite	−0.00 ± 0.87	0.00 ± 1.13	0.976	−0.01	Mann–Whitney U

Data are presented as mean ± SD. Neurocognitive measures were standardised as *z* scores across the full analytical sample (*N* = 86) after score directions were aligned so that higher values consistently represented better performance. Processing-speed composites were derived by averaging standardised scores for reversed TMT-A completion time, SDMT performance, and reversed Go/No-Go reaction time. Executive-function composites were derived by averaging standardised scores for reversed TMT-B completion time, reversed Stroop interference time, and Go/No-Go response accuracy. Memory composites were derived by averaging standardised RAVLT immediate recall, delayed recall, recognition, and interference recall scores. Working-memory composites were represented by the standardised Digit Span score. Higher composite scores indicate better neurocognitive performance. RAVLT, Rey Auditory Verbal Learning Test; SDMT, Symbol Digit Modalities Test; TMT, Trail Making Test.

**Figure 2:**
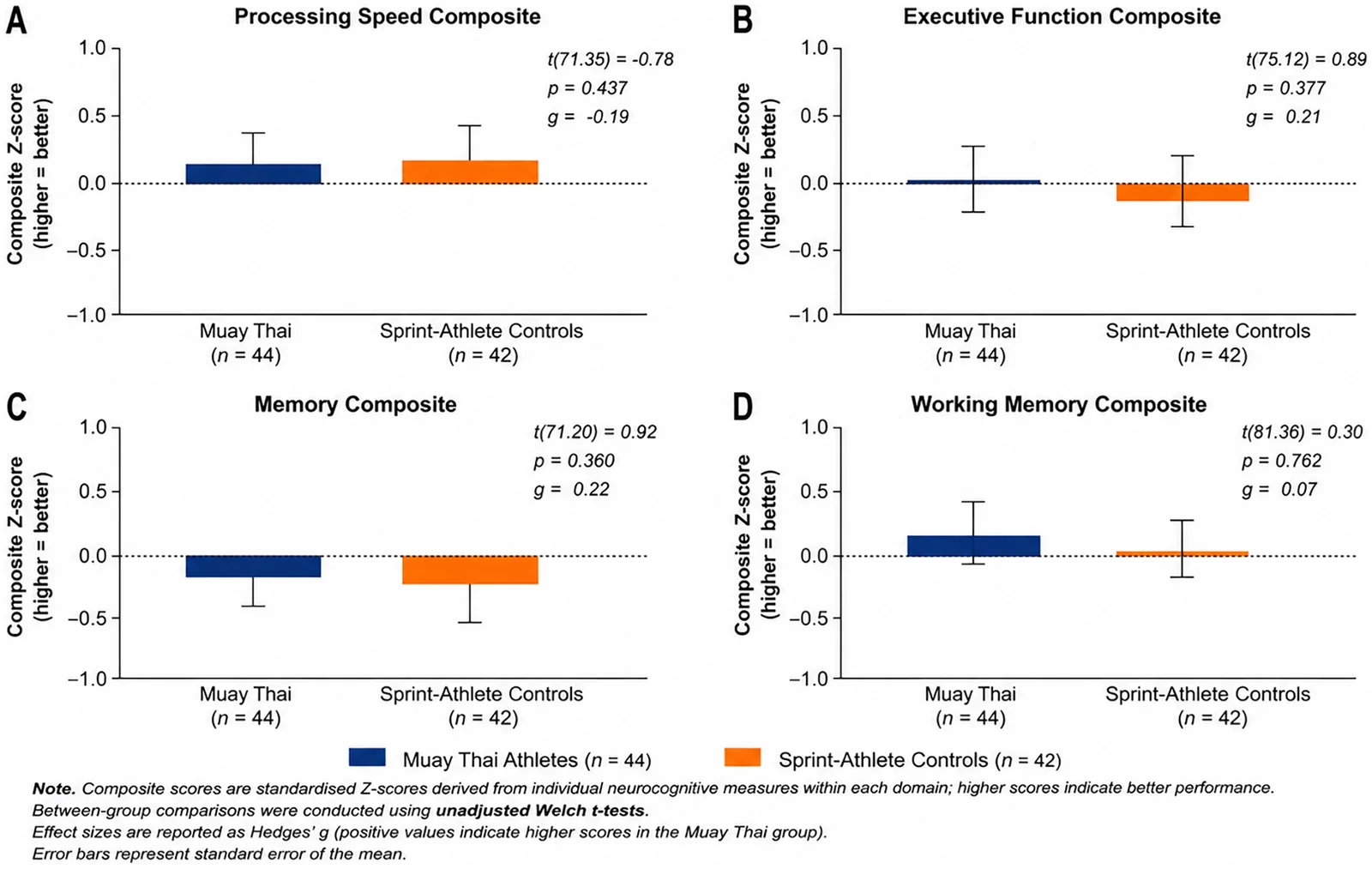
Composite neurocognitive-domain indicators in elite Muay Thai athletes and sprint-athlete controls selected for group-level comparability. Composite *z*-scores were derived from pooled neurocognitive task-level outcomes after alignment of score directionality, with higher values indicating better neurocognitive performance. Data are presented as box-and-whisker plots with individual participant data points. Between-group comparisons shown in the figure are unadjusted and should be distinguished from the covariate-adjusted ANCOVA models reported in the text and Supplementary Table S2.

Covariate-adjusted ANCOVA models controlling for age, years of education, and years of sport-specific training likewise showed no statistically significant group effects for processing speed, *F*(1, 81) = 0.31, *p* = 0.579, partial *η*^2^ = 0.004; executive function, *F*(1, 81) = 0.08, *p* = 0.777, partial *η*^2^ = 0.001; memory, *F*(1, 81) = 2.97, *p* = 0.088, partial *η*^2^ = 0.035; or working memory, *F*(1, 81) = 0.01, *p* = 0.942, partial *η*^2^ < 0.001. Tests of the Group×Age, Group×Education, and Group×Training Years interactions were non-significant across all four domain-level models (all *p* > 0.05), providing no evidence of violation of the homogeneity-of-regression-slopes assumption.

Model diagnostics indicated no problematic multicollinearity (VIFs=1.11–2.36; tolerance=0.42–0.90), and residual normality was acceptable across the four models. Evidence of heteroscedasticity was observed for the working-memory model; however, a heteroscedasticity-consistent (HC3) sensitivity analysis yielded the same substantive conclusion, with no statistically significant adjusted group effect (*p* = 0.944). Full adjusted-model estimates and diagnostic results are presented in Supplementary Table S2.

### 3.4 Repetitive head-impact exposure profile in Muay Thai athletes

Descriptive RHI exposure characteristics for the Muay Thai cohort are presented in Table 5. Athletes reported a mean of 4.84 ± 1.57 years of structured Muay Thai training and 25.00 ± 4.31 competitive fights. Self-reported head-injury counts and the cumulative RHI exposure score are also summarised in Table 5.

**Table 5:** Repetitive head-impact exposure characteristics in elite Muay Thai athletes (mean ± SD).

RHI variable	Mean ± SD	Min	Max
Training years	4.84 ± 1.57	3.0	9.0
Number of fights	25.00 ± 4.31	17.0	34.0
Head-injury history (self-reported events)	0.61 ± 0.75	0.0	3.0
Cumulative RHI exposure score	0.00 ± 2.66	−3.84	7.91

The cumulative repetitive head-impact (RHI) exposure score was calculated as the sum of standardised *z* scores for years of structured Muay Thai training, number of competitive fights, and self-reported number of head-injury events within the Muay Thai cohort. Higher scores indicate greater estimated cumulative exposure. RHI, repetitive head impact.

The cumulative RHI exposure score was calculated as the sum of standardised *z* scores for years of structured Muay Thai training, number of competitive fights, and self-reported number of head-injury events.

### 3.5 Associations between cumulative repetitive head-impact exposure and neurocognitive outcomes

Associations between cumulative RHI exposure and neurocognitive outcomes within the Muay Thai cohort are presented in Table 6. Among the four domain-level indicators, cumulative RHI exposure was negatively associated with the executive-function composite (Spearman's *ρ* = −0.32, *p* = 0.036), indicating that higher cumulative RHI exposure scores were associated with lower executive-function composite scores. No statistically significant associations were observed for processing speed (*ρ* = −0.09, *p* = 0.543), memory (*ρ* = −0.01, *p* = 0.967), or working memory (*ρ* = 0.24, *p* = 0.124).

**Table 6:** Spearman correlations between cumulative repetitive head-impact exposure and neurocognitive outcomes in elite Muay Thai athletes.

Outcome	Spearman's *ρ*	*p*-value
Composite neurocognitive-domain indicators		
Processing speed composite	−0.09	0.543
Executive function composite	−0.32	0.036
Memory composite	−0.01	0.967
Working memory composite	0.24	0.124
Task-specific neurocognitive outcomes		
TMT-A completion time	−0.44	0.003
TMT-B completion time	−0.41	0.006
Digit Span	0.24	0.124
Stroop interference time	0.56	<0.001
SDMT	−0.30	0.051
RAVLT Immediate Recall	0.02	0.884
RAVLT Delayed Recall	0.08	0.612
RAVLT Recognition	−0.02	0.886
RAVLT Interference Recall	−0.06	0.716
Go/No-Go reaction time	0.27	0.082
Go/No-Go response accuracy	−0.28	0.064

Values represent Spearman's ρ coefficients calculated within the elite Muay Thai cohort (*n* = 44). The cumulative repetitive head-impact (RHI) exposure score was calculated as the sum of standardised *z* scores for years of structured Muay Thai training, number of competitive fights, and self-reported number of head-injury events. Composite neurocognitive-domain indicators were derived from component neurocognitive measures standardised across the full analytical sample (*N* = 86), with score directions aligned so that higher composite values consistently represented better neurocognitive performance. For task-specific outcomes, shorter completion times indicate better performance on TMT-A, TMT-B, and Stroop interference, whereas shorter reaction times indicate faster Go/No-Go responses; higher scores indicate better performance on the SDMT, Digit Span, RAVLT outcomes, and Go/No-Go response accuracy. Analyses were exploratory, and *p*-values were not adjusted for multiple comparisons; statistically significant associations should therefore be interpreted as hypothesis-generating. RHI, repetitive head impact; TMT, Trail Making Test; SDMT, Symbol Digit Modalities Test; RAVLT, Rey Auditory Verbal Learning Test.

At the task-specific level, cumulative RHI exposure was associated with TMT-A completion time (*ρ* = −0.44, *p* = 0.003), TMT-B completion time (*ρ* = −0.41, *p* = 0.006), and Stroop interference time (*ρ* = 0.56, *p* < 0.001). No statistically significant associations were observed for SDMT performance (*ρ* = −0.30, *p* = 0.051), Digit Span (*ρ* = 0.24, *p* = 0.124), Go/No-Go reaction time (*ρ* = 0.27, *p* = 0.082), Go/No-Go response accuracy (*ρ* = −0.28, *p* = 0.064), or any RAVLT outcome (all *p* > 0.05). The statistically significant exploratory associations are illustrated in Figure 3.

**Figure 3:**
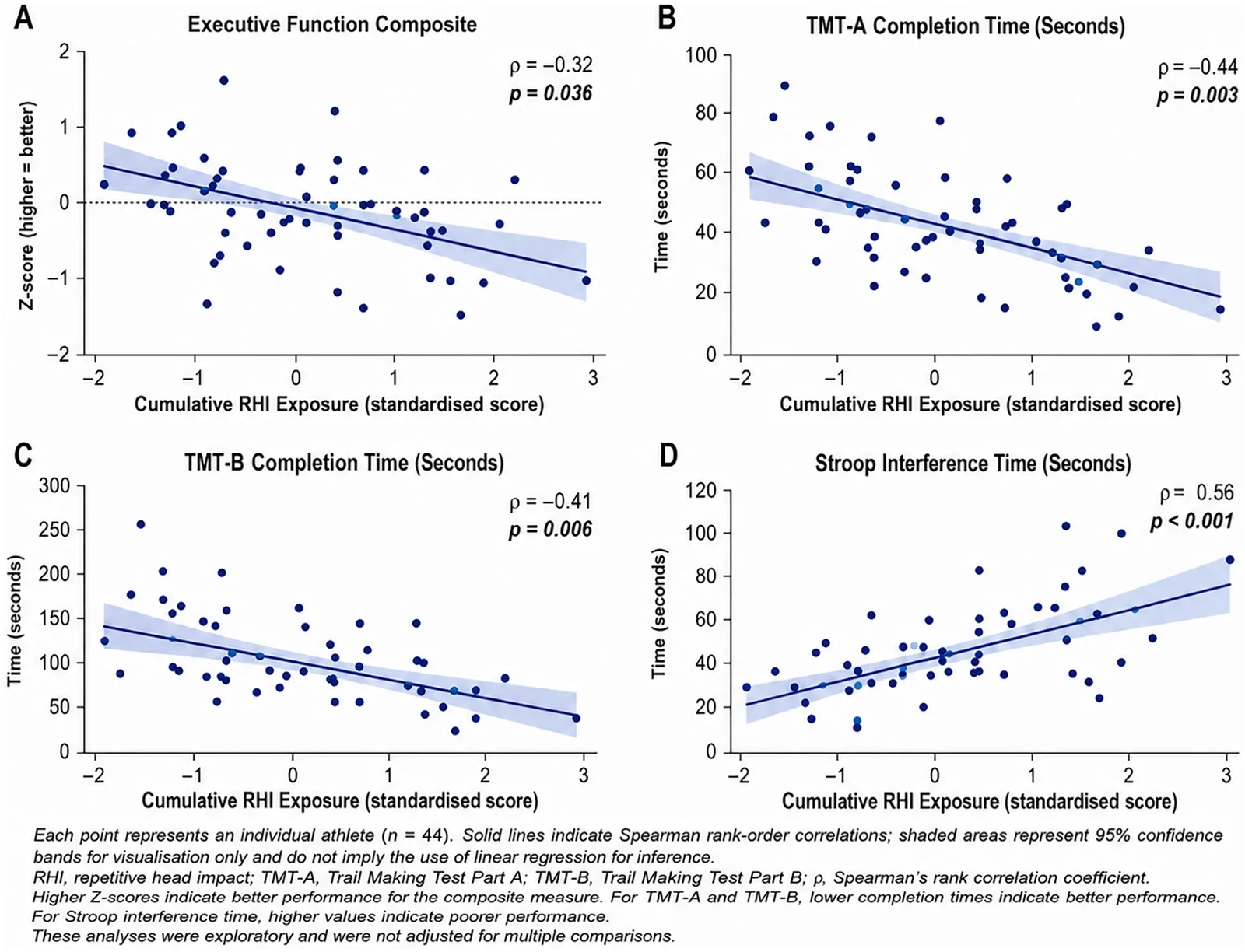
Exploratory associations between cumulative repetitive head-impact (RHI) exposure and selected neurocognitive outcomes in elite Muay Thai athletes (*n* = 44). Higher cumulative RHI exposure scores were associated with lower executive-function composite scores **(A)**, shorter TMT-A completion times **(B)**, shorter TMT-B completion times **(C)**, and longer Stroop interference times **(D)** Higher executive-function composite scores indicate better neurocognitive performance; shorter TMT-A and TMT-B completion times indicate better task performance, whereas shorter Stroop interference times indicate better performance. Spearman's *ρ* and unadjusted *p*-values are shown. These analyses were exploratory and were not adjusted for multiple comparisons. RHI, repetitive head impact; TMT-A, Trail Making Test Part A; TMT-B, Trail Making Test Part B.

Full exploratory correlations between the individual RHI exposure indicators and neurocognitive outcomes are presented in Supplementary Table S3. As prespecified for these hypothesis-generating analyses, the reported *p*-values were not adjusted for multiple comparisons; accordingly, statistically significant associations should be interpreted cautiously as exploratory findings.

## 4 Discussion

### 4.1 Group-level neurocognitive performance

Contrary to the study hypothesis, elite Muay Thai athletes did not demonstrate significantly poorer task-specific or domain-level neurocognitive performance than sprint-athlete controls. Executive function, processing speed, memory, and working memory were broadly comparable between groups. This pattern differs from findings in some collision- and combat-sport studies that have reported poorer performance on selected neurocognitive measures in association with repetitive head-impact exposure or longer exposure histories (2, 7, 32). Differences in comparator selection may partly explain these contrasting findings. Whereas some previous studies compared contact-sport athletes with non-athletic, non-contact, or differently trained comparison groups, the present study used highly trained sprint athletes selected for group-level comparability in demographic and training characteristics (14, 15). This athletic-control design was intended to reduce confounding from characteristics associated with long-term participation in high-performance sport, although residual confounding remains possible.

The comparable performance of the two groups should also be considered in relation to cognitive characteristics associated with sustained physical and sport training. Regular physical activity and martial-arts training have been associated with better executive-function performance, attentional regulation, inhibitory control, and cognitive processing efficiency (9–13). Such training-related characteristics may contribute to neurocognitive performance in both athletic groups and complicate attribution of cross-sectional differences specifically to RHI exposure. Neurocognitive performance in elite combat-sport athletes may therefore reflect the combined influence of sport-specific exposure and long-term athletic training rather than RHI exposure alone.

The relatively young age and active competitive status of the present cohort provide important context for the absence of broad between-group differences. Studies involving athletes with longer competitive careers or greater lifetime exposure have reported poorer performance on selected executive-function and processing-speed measures, whereas findings among younger or actively competing athletes have been less consistent (7, 32, 33). For example, active professional male footballers have shown largely preserved overall neurocognitive function, although selected attentional outcomes were associated with concussion history (34). Differences across studies may reflect variation in athlete age, competitive history, cumulative exposure burden, sport-specific impact characteristics, neurocognitive outcome selection, and approaches used to quantify head-impact exposure. Accordingly, the present findings should not be interpreted to mean that RHIs lack potential neurocognitive relevance. Rather, broad group-level differences in neurocognitive performance were not evident in this sample of young, actively competing Muay Thai athletes compared with athletic controls matched at the group level.

### 4.2 Associations between cumulative repetitive head-impact exposure and neurocognitive outcomes

Within the Muay Thai cohort, exploratory analyses identified an association between cumulative RHI exposure and the executive-function composite, together with task-specific associations involving TMT-A, TMT-B, and Stroop interference. This pattern partially overlaps with previous contact- and combat-sport research examining executive control, cognitive flexibility, inhibitory control, and processing speed in relation to cumulative head-impact exposure (7, 15). However, the associations were selective rather than uniform: processing-speed, memory, and working-memory composites were not associated with cumulative RHI exposure, and related individual measures did not consistently show corresponding associations. The findings therefore indicate limited covariation between estimated cumulative RHI exposure and selected neurocognitive outcomes rather than generalised neurocognitive impairment.

The directions of the task-specific associations further argue against a uniform exposure-related pattern of poorer performance. Higher cumulative RHI exposure scores were associated with shorter TMT-A and TMT-B completion times but longer Stroop interference times. Because shorter completion times indicate better performance on TMT-A and TMT-B, whereas longer Stroop interference times indicate poorer performance, these associations point in opposing performance-related directions. This inconsistency was also evident within the domain-level indicators: the executive-function composite was negatively associated with cumulative RHI exposure, yet its component measures showed shorter TMT-B completion time, longer Stroop interference time, and no association with Go/No-Go response accuracy. Similarly, TMT-A was associated with cumulative exposure, whereas the processing-speed composite and its other component measures were not. These exploratory findings therefore do not support a uniform or preferential domain-specific effect.

Interpretation is further constrained by methodological differences across the RHI literature, including athlete age, competitive history, exposure burden, sport-specific impact characteristics, neurocognitive outcome selection, and approaches to head-impact quantification. In the present study, the cumulative RHI score was derived from retrospectively self-reported proxy indicators rather than direct biomechanical monitoring, the correlations were not adjusted for multiple comparisons, and the cross-sectional design precludes temporal or causal inference. Exposure misclassification and Type I error are therefore possible, and the observed associations should be regarded as hypothesis-generating rather than confirmatory.

These findings do not support the use of individual neurocognitive measures, domain-level indicators, or the cumulative RHI score as stand-alone screening, diagnostic, return-to-play, or clinical risk-assessment tools. The identified outcomes may serve as candidate measures for prospective research and, where neurocognitive assessment is incorporated into athlete-health settings, should be interpreted alongside relevant clinical information and training, competition, and head-injury histories. Prospective longitudinal studies incorporating objective head-impact monitoring and repeated neurocognitive assessment are needed before these measures can inform routine surveillance or individual athlete-management decisions.

### 4.3 Methodological strengths and limitations

The athletic-control design with group-level matching, multidimensional neurocognitive battery, and complementary task-specific, domain-level, and covariate-adjusted analyses provided multiple perspectives on neurocognitive performance while reducing, but not eliminating, potential confounding associated with differences in demographic and training characteristics.

Several limitations should be considered. The cross-sectional design precludes temporal or causal inference, and the retrospectively self-reported proxy-based RHI measure was susceptible to recall bias and exposure misclassification and could not quantify the frequency, magnitude, location, or biomechanical characteristics of individual head impacts. Group-level matching did not ensure individual equivalence, and covariate adjustment could not account for all measured or unmeasured confounding. Generalisability is limited by the male sample and specific competitive and geographic context, while restricted variability or ceiling effects may have reduced sensitivity to subtle neurocognitive differences. The study-specific domain indicators were complementary summary measures rather than independently validated neuropsychological composites, with working memory represented by Digit Span alone. Finally, the exploratory within-Muay Thai correlations were unadjusted for multiple comparisons and therefore susceptible to Type I error; these findings should be regarded as hypothesis-generating. Prospective longitudinal studies incorporating objective head-impact monitoring and more diverse athlete samples are needed to assess reproducibility and neurocognitive change with accumulating exposure.

## 5 Conclusions

Elite Muay Thai athletes did not demonstrate significant group-level differences in task-specific or domain-level neurocognitive performance compared with sprint-athlete controls selected for group-level comparability. Within the Muay Thai cohort, exploratory analyses identified associations between cumulative repetitive head-impact (RHI) exposure and the executive-function composite, TMT-A completion time, TMT-B completion time, and Stroop interference time. These associations were selective and directionally inconsistent with respect to performance across the broader neurocognitive battery; accordingly, they should not be interpreted as evidence of generalised neurocognitive impairment or a uniform exposure-related pattern of poorer performance. Given the cross-sectional design, retrospective proxy-based exposure assessment, and unadjusted exploratory comparisons, the findings should be regarded as hypothesis-generating rather than causal or confirmatory. Prospective longitudinal studies incorporating objective head-impact monitoring and repeated neurocognitive assessment are needed to determine whether these associations are reproducible and how neurocognitive performance changes with accumulating exposure over time.

## Data Availability

The raw data supporting the conclusions of this article will be made available by the authors, without undue reservation.
