# Supplementary material for: Sport-related neurocognitive performance and repetitive head-impact exposure in elite Muay Thai athletes: a matched athletic-control study
Source: Front Sports Act Living. 2026 Oct 14;8:1908733. doi: 10.3389/fspor.2026.1908733 (PMC13713806; doi:10.3389/fspor.2026.1908733)
Supplement: Supplementary file 1 [file Table1.docx]

**Supplementary Material**

**Sport-related neurocognitive performance and repetitive head-impact exposure in elite Muay Thai athletes: a matched athletic-control study**

**Supplementary Table S1. Normality assessment of neurocognitive variables**

| **Variable** | **Shapiro–Wilk W** | **p-value** | **Skewness** | **Kurtosis** |
| --- | --- | --- | --- | --- |
| TMT-A | 0.97 | 0.081 | -0.42 | -0.11 |
| TMT-B | 0.98 | 0.214 | 0.31 | -0.27 |
| Digit Span | 0.95 | 0.019 | -0.61 | 0.44 |
| Stroop Test | 0.98 | 0.146 | 0.22 | -0.33 |
| SDMT | 0.96 | 0.041 | -0.49 | 0.18 |
| RAVLT Immediate | 0.95 | 0.022 | -0.70 | 0.59 |
| RAVLT Delayed/Recall | 0.97 | 0.067 | -0.36 | -0.15 |
| Reaction Time | 0.98 | 0.173 | 0.18 | -0.41 |
| Accuracy | 0.94 | 0.011 | -0.83 | 0.92 |
| Processing Speed Composite | 0.98 | 0.281 | -0.12 | -0.38 |
| Executive Function Composite | 0.97 | 0.119 | -0.21 | -0.19 |
| Memory Composite | 0.96 | 0.037 | -0.46 | 0.11 |
| Working Memory Composite | 0.95 | 0.028 | -0.58 | 0.36 |

*Note*. Variables with Shapiro–Wilk p-values < 0.05 were considered to show departures from normality and were analysed using non-parametric procedures where appropriate. Abbreviations: TMT, Trail Making Test; SDMT, Symbol Digit Modalities Test; RAVLT, Rey Auditory Verbal Learning Test.

**Supplementary Table S2. Covariate-adjusted ANCOVA models and diagnostic assessments for composite neurocognitive-domain indicators**

**Panel A. Covariate-adjusted ANCOVA models**

| **Outcome** | **Covariates** | ***F*(1, 81)** | ***p*** | **Partial η²** |
| --- | --- | --- | --- | --- |
| Processing speed composite | Age, education, training years | 0.31 | 0.579 | 0.004 |
| Executive function composite | Age, education, training years | 0.08 | 0.777 | 0.001 |
| Memory composite | Age, education, training years | 2.97 | 0.088 | 0.035 |
| Working memory composite | Age, education, training years | 0.01 | 0.942 | <0.001 |

Note. Separate ANCOVA models were fitted for each composite neurocognitive-domain indicator, with group (Muay Thai vs sprint-athlete control) entered as the fixed factor and age, years of education, and years of sport-specific training entered as covariates. Composite indicators were derived from neurocognitive measures standardised across the full analytical sample (N = 86), with score directions aligned so that higher composite values consistently represented better neurocognitive performance. Reported F, p, and partial η² values correspond to the adjusted group effect in each model. No statistically significant adjusted group effects were observed across the four neurocognitive domains.

**Panel B. ANCOVA model diagnostics**

| **Diagnostic assessment** | **Result** | **Interpretation** |
| --- | --- | --- |
| Multicollinearity | VIF = 1.11–2.36; tolerance = 0.42–0.90 | No evidence of problematic multicollinearity |
| Group × Age interactions | All *p* > 0.05 | No evidence of violation of the homogeneity-of-regression-slopes assumption |
| Group × Education interactions | All *p* > 0.05 | No evidence of violation of the homogeneity-of-regression-slopes assumption |
| Group × Training Years interactions | All *p* > 0.05 | No evidence of violation of the homogeneity-of-regression-slopes assumption |
| Residual normality | Shapiro–Wilk tests: all *p* > 0.05; Q–Q plots showed no material departures | No material departure from residual normality |
| Homoscedasticity | No material evidence of heteroscedasticity for processing speed, executive function, or memory; evidence of heteroscedasticity for working memory | Working-memory model examined using HC3 sensitivity analysis |
| Influential observations | No Cook’s distance > 1 | No highly influential observations identified |
| Working-memory HC3 sensitivity analysis | *p* = 0.944 | Adjusted group effect remained non-significant |

Note. Diagnostic assessments correspond to the separate ANCOVA models reported in Panel A, with group (Muay Thai vs sprint-athlete control) entered as the fixed factor and age, years of education, and years of sport-specific training entered as covariates. Composite neurocognitive-domain indicators were derived from measures standardised across the full analytical sample (N = 86), with score directions aligned so that higher composite values consistently represented better neurocognitive performance. Multicollinearity was evaluated using variance inflation factors (VIFs), tolerance values, and correlations among covariates. Homogeneity of regression slopes was assessed using Group × Age, Group × Education, and Group × Training Years interaction terms. Residual normality was evaluated using Shapiro–Wilk tests and inspection of Q–Q plots, while homoscedasticity was assessed using residual diagnostics and Levene’s test. Standardised residuals, leverage values, and Cook’s distance were inspected to identify potentially influential observations. Evidence of heteroscedasticity was identified for the working-memory model; therefore, a heteroscedasticity-consistent (HC3) sensitivity analysis was performed. The adjusted group effect for working memory remained non-significant (p = 0.944), leaving the substantive interpretation unchanged.

**Supplementary Table S3. Exploratory correlations between repetitive head-impact exposure variables and neurocognitive outcomes**

| **Outcome** | **Training years** | **Number of fights** | **Number of head-injury events** | **Cumulative RHI score** |
| --- | --- | --- | --- | --- |
| **Composite neurocognitive-domain indicators** |  |  |  |  |
| Processing speed composite | −0.05 | −0.06 | −0.17 | −0.09 |
| Executive function composite | −0.16 | −0.30 | −0.35* | −0.32* |
| Memory composite | 0.09 | 0.03 | −0.07 | −0.01 |
| Working memory composite | 0.34* | 0.15 | 0.16 | 0.24 |
| **Task-specific neurocognitive outcomes** |  |  |  |  |
| TMT-A | −0.42** | −0.46** | −0.28 | −0.44** |
| TMT-B | −0.47** | −0.35* | −0.33* | −0.41** |
| Digit Span | 0.34* | 0.15 | 0.16 | 0.24 |
| Stroop interference time | 0.45** | 0.53*** | 0.44** | 0.56*** |
| SDMT | −0.16 | −0.26 | −0.42** | −0.30 |
| RAVLT Immediate Recall | 0.19 | 0.03 | −0.03 | 0.02 |
| RAVLT Delayed Recall | 0.04 | 0.07 | 0.01 | 0.08 |
| RAVLT Recognition | −0.02 | 0.03 | −0.06 | −0.02 |
| RAVLT Interference Recall | 0.07 | −0.04 | −0.05 | −0.06 |
| Go/No-Go reaction time | 0.21 | 0.26 | 0.20 | 0.27 |
| Go/No-Go response accuracy | −0.24 | −0.21 | −0.37* | −0.28 |

Note. Values represent Spearman’s ρ coefficients calculated within the Muay Thai cohort (n = 44). The cumulative repetitive head-impact (RHI) exposure score was calculated as the sum of standardised z scores for years of structured Muay Thai training, number of competitive fights, and self-reported number of head-injury events. Composite neurocognitive-domain indicators were derived from component neurocognitive measures standardised across the full analytical sample (N = 86), with score directions aligned so that higher composite values consistently represented better neurocognitive performance. Analyses were exploratory, and p-values were not adjusted for multiple comparisons. **Significance symbols therefore refer to unadjusted** p**-values: ***p **< 0.05; ****p **< 0.01; *****p **< 0.001.** Abbreviations: RHI, repetitive head impact; TMT, Trail Making Test; SDMT, Symbol Digit Modalities Test; RAVLT, Rey Auditory Verbal Learning Test.
